# A novel β-glucosidase from *Saccharophagus degradans* 2-40^T^ for the efficient hydrolysis of laminarin from brown macroalgae

**DOI:** 10.1186/s13068-018-1059-2

**Published:** 2018-03-14

**Authors:** Dong Hyun Kim, Do Hyoung Kim, Sang-Hyun Lee, Kyoung Heon Kim

**Affiliations:** Department of Biotechnology, Graduate School, Korea University, Seoul, 02841 South Korea

**Keywords:** β-Glucosidase, Laminarin, Laminaribiose, Transglycosylation, Brown macroalgae

## Abstract

**Background:**

Laminarin is a potential biomass feedstock for the production of glucose, which is the most preferable fermentable sugar in many microorganisms by which it can be converted to biofuels and bio-based chemicals. Also, laminarin is a good resource as functional materials because it consists of β-1,3-glucosidic linkages in its backbone and β-1,6-glucosidic linkages in its branches so that its oligosaccharides driven from laminarin have a variety of biological activities. It is industrially important to be able to produce laminarioligosaccharides as well as glucose from laminarin by a single enzyme because the enzyme cost accounts for a large part of bio-based products. In this study, we investigated the industrial applicability of Bgl1B, a unique β-glucosidase from *Saccharophagus degradans* 2-40^T^, belonging to the glycoside hydrolase family 1 (GH1) by characterizing its activity of hydrolyzing laminarin under various conditions.

**Results:**

Bgl1B was cloned and overexpressed in *Escherichia coli* from *S. degradans* 2-40^T^, and its enzymatic activity was characterized. Similar to most of β-glucosidases in GH1, Bgl1B was able to hydrolyze a variety of disaccharides having different β-linkages, such as laminaribiose, cellobiose, gentiobiose, lactose, and agarobiose, by cleaving β-1,3-, β-1,4-, and β-1,6-glycosidic linkages. However, Bgl1B showed the highest specific activity toward laminaribiose with a β-1,3-glycosidic linkage. In addition, it was able to hydrolyze laminarin, one of the major polysaccharides in brown macroalgae, into glucose with a conversion yield of 75% of theoretical maximum. Bgl1B also showed transglycosylation activity by producing oligosaccharides from laminarin and laminaribiose under a high mass ratio of substrate to enzyme. Furthermore, Bgl1B was found to be psychrophilic, exhibiting relative activity of 59–85% in the low-temperature range of 2–20 °C.

**Conclusions:**

Bgl1B can directly hydrolyze laminarin into glucose with a high conversion yield without leaving any oligosaccharides. Bgl1B can exhibit high enzymatic activity in a broad range of low temperatures (2–20 °C), which is advantageous for establishing energy-efficient bioprocesses. In addition, under high substrate to enzyme ratios, Bgl1B can produce high-value laminarioligosaccharides via its transglycosylation activity. These results show that Bgl1B can be an industrially important enzyme for the production of biofuels and bio-based chemicals from brown macroalgae.

**Electronic supplementary material:**

The online version of this article (10.1186/s13068-018-1059-2) contains supplementary material, which is available to authorized users.

## Background

With increasing concern over global warming, renewable biomass has received much attention owing to its sustainability. Lignocellulosic biomass has long been considered a top priority renewable biomass feedstock for producing fermentable sugars because of its abundance and low price. However, the recalcitrance of lignocellulosic biomass has hindered its easy use as a biomass feedstock for the production of bio-based chemicals. Recently, marine macroalgae have emerged as potent alternatives to lignocellulosic biomass because of their several advantages, such as no recalcitrance, high carbohydrate contents, short life cycles and no requirement of arable land or fertilizers for their cultivation [1].

Among marine macroalgae, more than 70 million tons of brown macroalgae are harvested annually worldwide [2]. The main carbohydrate components of brown macroalgae are alginate and glucan, and glucan mainly occurs as laminarin, a glucose polysaccharide with β-1,3-glucosidic linkages in its backbone and β-1,6-glucosidic linkages in its branches [3]. Among brown macroalgae, *Laminarina*, *Saccharina*, and *Fucus* spp. have high laminarin contents (max. 62% in their dry weights) [4]. Because laminarin is composed of glucose, it is an ideal substrate from macroalgal biomass for producing glucose as a fermentable sugar. Although there have been many studies on the utilization of brown macroalgae as feedstocks for the production of bio-based chemicals [5–7], most of them are related to depolymerization of alginate, not laminarin. In some cases of laminarin depolymerization, the studies explored β-1,3-glucanases that produce mainly oligosaccharides and a small amount of glucose simultaneously [8–10]. However, there have been no studies on β-glucosidases that catalyze the production of glucose as the sole product. Therefore, it is necessary to construct a simple and efficient enzymatic saccharification system to produce glucose from laminarin.

In this study, we have cloned, expressed, and characterized a novel β-glucosidase, Bgl1B, from a marine bacterium, *Saccharophagus degradans*, 2-40^T^. In addition, the saccharification capability of this enzyme was investigated under various conditions. To our knowledge, Bgl1B is a novel β-glucosidase having the unique laminarin-hydrolyzing capability. This is the first report that a single enzyme can hydrolyze laminarin and produce glucose as the sole product with a high product yield.

## Results

### Overexpression and purification of recombinant Bgl1B

**Fig. 1:**
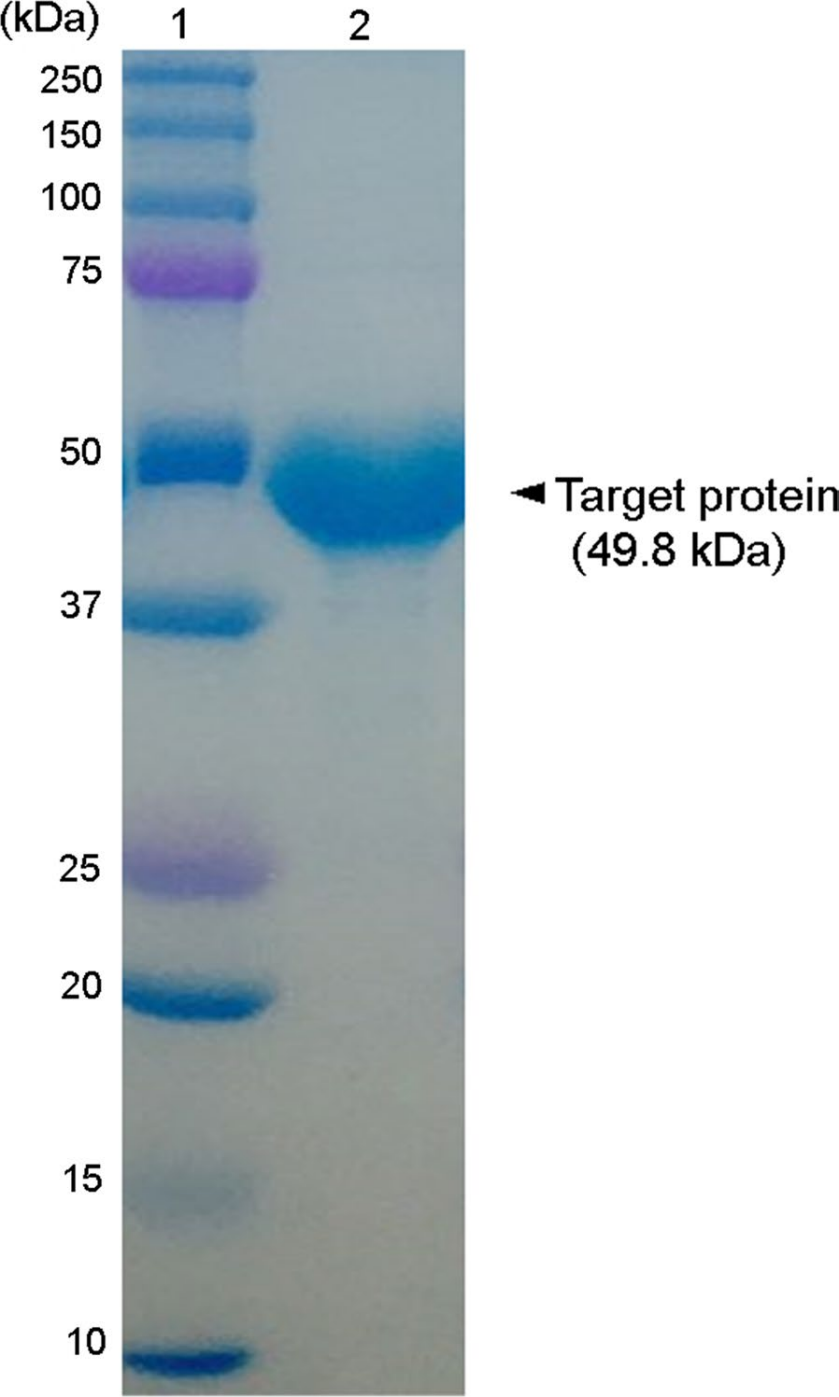
SDS-PAGE analysis of the purified recombinant Bgl1B. Lanes: 1, protein marker; 2, Bgl1B purified by His-tag affinity chromatography

To investigate the enzyme activity of Bgl1B, the plasmid pET21a–Bgl1B was constructed under the control of T7 promoter with the sequence encoding a 6-His tag at the C terminus. The plasmids were introduced into *Escherichia coli* BL21(DE3). To minimize inclusion body formation, the recombinant *E. coli* was grown to reach an optical density at 600 nm of 0.5 in LB media at 37 °C, and protein expression was induced with 0.5 mM IPTG at 16 °C for 16 h. The recombinant Bgl1B protein with a theoretical molar mass of 49.8 kDa was successfully expressed in soluble form and purified for further experiments (Fig. 1).

### Optimal pH and temperature for Bgl1B

**Fig. 2:**
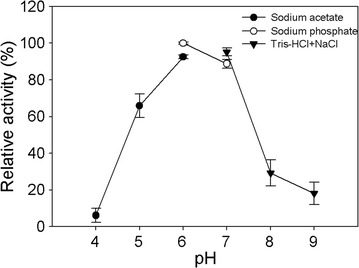
Effect of pH on the activity of Bgl1B. The enzymatic reactions were performed using buffers of different pHs (pH 4.0–9.0) at 40 °C for 15 min

To determine the optimal pH and temperature of Bgl1B, 0.06 U of Bgl1B was incubated with 0.1% (w/v) cellobiose as the substrate at various pHs (pH 4.0–9.0) and temperatures (2–60 °C). The highest enzymatic activity was obtained at pH 6.0 in 20 mM sodium phosphate buffer (Fig. 2). At both pH 4.0 in 20 mM sodium acetate buffer and over pH 8.0 in 20 mM Tris–HCl–NaCl buffer, the relative activities of Bgl1B decreased to the values lower than 30%.

**Fig. 3:**
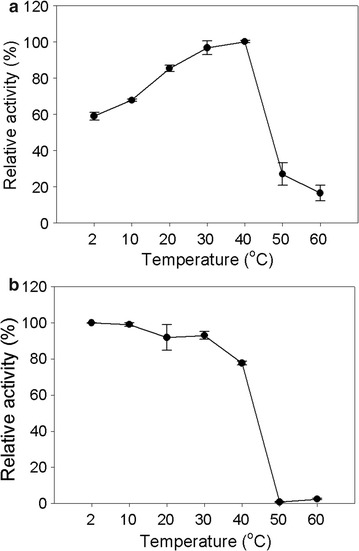
Effect of temperature on the activity of Bgl1B. The optimal temperature (**a**) and thermal stability (**b**) of Bgl1B were determined. The enzymatic reactions were performed at different temperatures (20–60 °C) for 15 min in 20 mM sodium phosphate buffer (pH 6.0)

The optimal temperature of Bgl1B was 40 °C at pH 6.0, and Bgl1B maintained approximately 83% of its relative activity at 20 °C (Fig. 3a). Interestingly, even at extremely low temperatures (2–10 °C), more than 58% of relative activity was maintained. However, at 50 °C, the relative activity decreased sharply to approximately 27% (Fig. 3a). In the thermal stability test, the relative activity of Bgl1B was maintained at 80% after 1 h pre-incubation at 40 °C. However, after the pre-incubation at higher than 40 °C, Bgl1B was not active at all (Fig. 3b).

### Effect of metal ions on the activity of Bgl1B

Metal ions can act as cofactors or inhibitors of enzymes. To examine the effect of metal ions on the activity of Bgl1B, enzyme assay was performed in the presence of various metal ions (10 mM) with 0.1% (w/v) cellobiose as the substrate (Table 1). The activity of Bgl1B was not noticeably affected by Mg^2+^, Ni^2+^, or Co^2+^ by maintaining more than 90% of relative activity. However, in the presence of Mn^2+^, the relative activity of Bgl1B significantly decreased to 53%. In the presence of Cu^2+^ or Fe^2+^, the relative activity of Bgl1B decreased to the values lower than 10%.

**Table 1:** Effect of various metal ions on the activity of Bgl1B

Metal ion	Relative enzymatic activity (%)
None	100.0 ± 2.8
Mg^2+^	98.9 ± 2.3
Ca^2+^	82.0 ± 1.5
Mn^2+^	52.6 ± 2.1
Ni^2+^	90.3 ± 2.7
Cu^2+^	0.0 ± 0.0
Fe^2+^	8.2 ± 0.3
Co^2+^	95.7 ± 1.6

Metal ions including Mg^2+^, Ca^2+^, Mn^2+^, Ni^2+^, Cu^2+^, Fe^2+^, and Co^2+^ act as cofactors or inhibitors of certain enzymes. There are several studies that Cu^2+^ or Fe^2+^ inhibits certain enzymes [8].

### Substrate specificity of Bgl1B

**Fig. 4:**
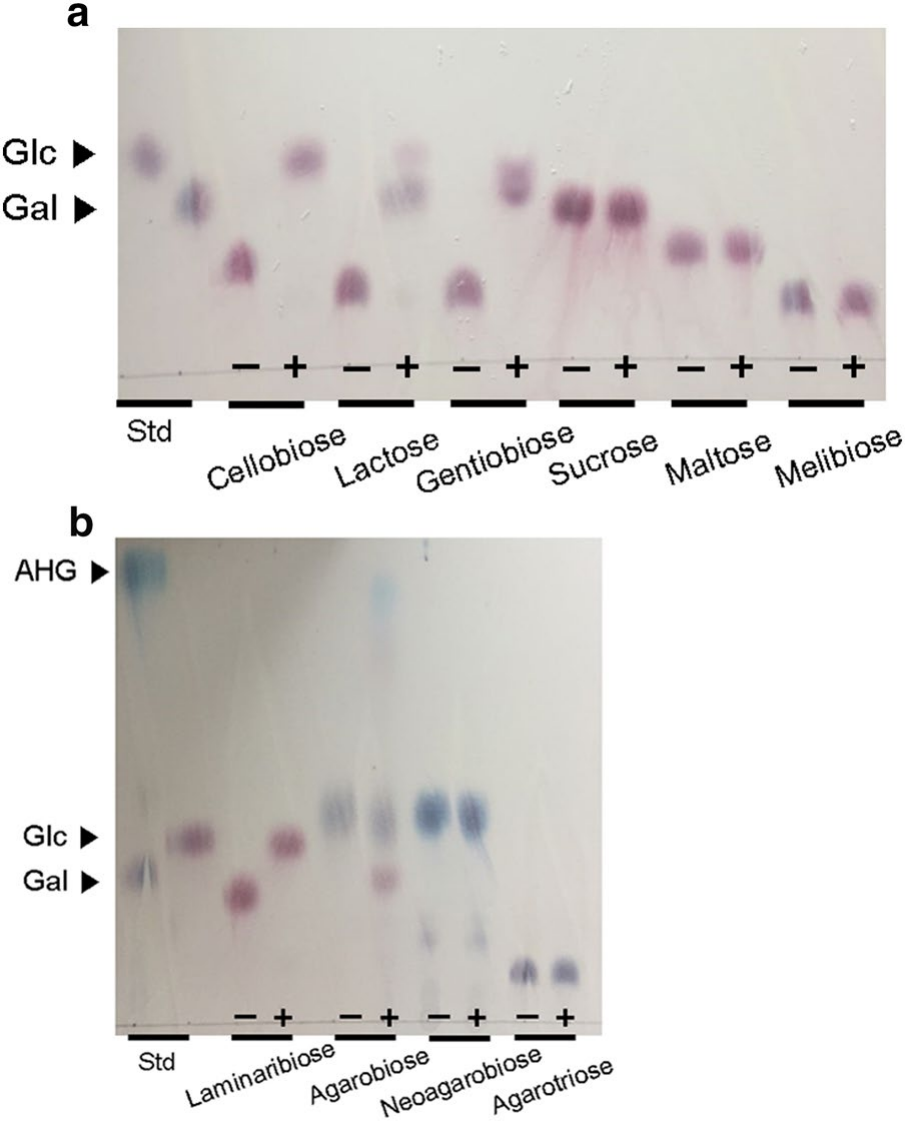
Substrate specificity of Bgl1B. TLC analysis of the reaction products obtained when recombinant Bgl1B was incubated for 1 h with **a** terrestrial biomass substrates and **b** marine macroalgal substrates. Glucose (Glc), galactose (Gal), and 3,6-anhydro-l-galactose (AHG) were used as the markers for the reaction products. Std, standard; −, substrate only; +, substrate incubated with Bgl1B

To examine the substrate specificity of Bgl1B, 0.2% (w/v) of several disaccharides possessing various types of glycosidic linkages were subjected to enzymatic reactions with Bgl1B (Fig. 4a, b). Among the group of terrestrial disaccharides, cellobiose (β-1,4-glycosidic linkage), lactose (β-1,4-glycosidic linkage), and gentiobiose (β-1,6-glycosidic linkage) were completely hydrolyzed into glucose, glucose and galactose, and glucose by Bgl1B, respectively (Fig. 4a). However, Bgl1B did not hydrolyze sucrose, maltose, and melibiose, which are the disaccharides possessing α-glycosidic linkages (Fig. 4a). Bgl1B also hydrolyzed marine-derived disaccharides such as laminaribiose having a β-1,3-glycosidic linkage and agarobiose having a β-1,4-glycosidic linkage, except for neoagarobiose having an α-1,3-glycosidic linkage (Fig. 4b). However, Bgl1B did not hydrolyze the β-1,4-linkage of agarotriose, which consists of two units of galactose and one unit of 3,6-anhydro-l-galactose in alternating β-1,4 and α-1,3-glycosidic linkages (Fig. 4b).

### Kinetic parameters of Bgl1B toward various substrates

To determine the kinetic parameters of Bgl1B toward various disaccharides such as laminaribiose, cellobiose, gentiobiose, lactose, and agarobiose, enzymatic reactions were performed at 40 °C in 20 mM sodium phosphate buffer (pH 6.0) with different substrate concentrations. The kinetic parameters of Bgl1B were determined using the Lineweaver–Burk plot (Table 2). The *V*_max_, *K*_m_, and *k*_cat_ values of Bgl1B for laminaribiose were 1.1 × 10^2^ U mg^−1^ protein, 9.9 × 10^−1^ mM, and 9.1 × 10^1^ s^−1^, respectively (Table 2). Bgl1B enzyme showed the highest catalytic efficiency (i.e., *k*_cat_/*K*_m_) of 9.2 × 10^1^ mM^−1^ s^−1^ for laminaribiose among other tested substrates (Table 2 and Additional file 1: Table S1). For laminaribiose, the *V*_max_, *K*_m_, and *k*_cat_ values of BglB1 were 259 times higher, 2.5 times lower, and 108 times higher than those of the commercial β-glucosidase, Novozyme 188, respectively (Table 2). Moreover, the catalytic efficiency of Bgl1B for laminaribiose was about 271 times higher than that of Novozyme 188. In addition, for cellobiose, the *V*_max_ and *k*_cat_ values of Bgl1B was 1.6 × 10^1^ U mg^−1^ protein and 1.3 × 10^1^ s^−1^, which was 4.0 and 1.6 times higher than those of Novozyme 188, respectively (Table 2). However, the *K*_m_ value of Bgl1B for cellobiose was 19.8 mM, which was 5.9 times higher than that of Novozyme 188. Therefore, the catalytic efficiency of Bgl1B for cellobiose was lower than that of Novozyme 188. For gentiobiose, lactose, and agarobiose, the catalytic efficiencies of Bgl1B were 6.6 × 10^−2^, 4.1 × 10^−2^, and 3.7 × 10^−3^ mM^−1^ s^−1^, respectively, which were lower than those for laminaribiose and cellobiose (Additional file 1: Table S1).

**Table 2:** Kinetic parameters of Bgl1B and Novozyme 188 in the hydrolysis of different types of substrates

Enzyme	Substrate	Glycosidic linkage	*V*_max_ (U^a^ mg^−1^ protein)	*K*_m_ (mM)	*k*_cat_ (s^−1^)	*k*_cat_/*K*_m_ (mM^−1^ s^−1^)
Bgl1B	Laminaribiose	β-1,3	1.1 × 10^2^	9.9 × 10^−1^	9.1 × 10^1^	9.2 × 10^1^
	Cellobiose	β-1,4	1.6 × 10^1^	2.0 × 10^1^	1.3 × 10^1^	6.5 × 10^−1^
Novozyme 188	Laminaribiose	β-1,3	4.2 × 10^−1^	2.5 × 10^0^	8.5 × 10^−1^	3.4 × 10^−1^
	Cellobiose	β-1,4	3.9 × 10^0^	3.4 × 10^0^	7.8 × 10^0^	2.3 × 10^0^

Kinetic parameters: each value of parameter is the mean of experimental triplicates

^a^One unit (U) of Bgl1B was defined as the amount of enzyme required to produce 1 μmol of glucose per min from 0.1% (w/v) cellobiose in 20 mM sodium phosphate buffer (pH 6.0) at 40 °C

### Mode of action of Bgl1B

**Fig. 5:**
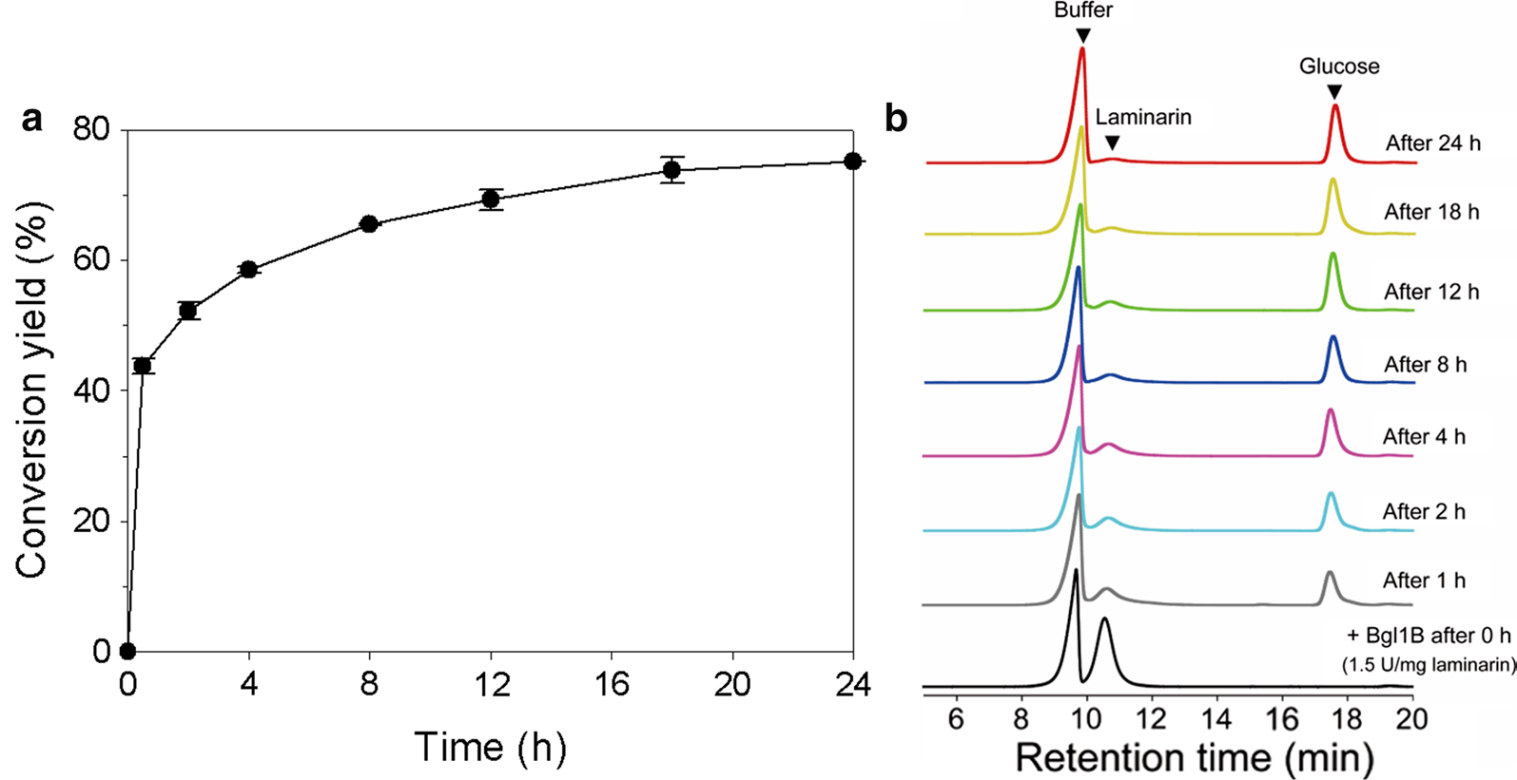
Hydrolysis activity of Bgl1B on laminarin substrate. **a** The conversion yield of Bgl1B on laminarin and **b** HPLC analyses of enzymatic reaction products. The enzymatic reactions were performed with 1.5 U mg^−1^ laminarin at 40 °C in 20 mM sodium phosphate (pH 6.0)

Laminaribiose is the main component of laminarin, a polysaccharide from brown macroalgae. Since Bgl1B showed the highest activity on laminaribiose among many disaccharides tested in this study, its ability to hydrolyze laminarin was investigated here. Interestingly, glucose was detected as a sole reaction product by HPLC analysis (Fig. 5b). The conversion yield of laminarin into glucose using 1.5 U mg^−1^ laminarin was 75% of theoretical maximum after 24 h (Fig. 5a). These results indicated that Bgl1B attacks the non-reducing end of laminarin. Phylogenetic analysis based on DNA sequence showed that Bgl1B is a β-glucosidase belonging to GH1 (Additional file 2: Figure S1). Meanwhile, since the laminarin from *Eisenia bicyclis*, a brown macroalga, consists of β-1,3- and β-1,6-glycosidic linkages in a ratio of 2:1 [11], it was not completely hydrolyzed by Bgl1B. These results indicated that Bgl1B hydrolyzes only β-1,3-glycosidic linkages in laminarin. Indeed, Bgl1B did not hydrolyze pustulan (Additional file 3: Figure S2), which is a glucose polysaccharide formed by β-1,6 glycosidic linkages. Based on the mode of action and the phylogenetic analysis of Bgl1B revealed above, Bgl1B was suggested to be mainly an exo-type β-1,3-glucanase hydrolyzing only β-1,3-glucan but not β-1,6-glucan.

**Fig. 6:**
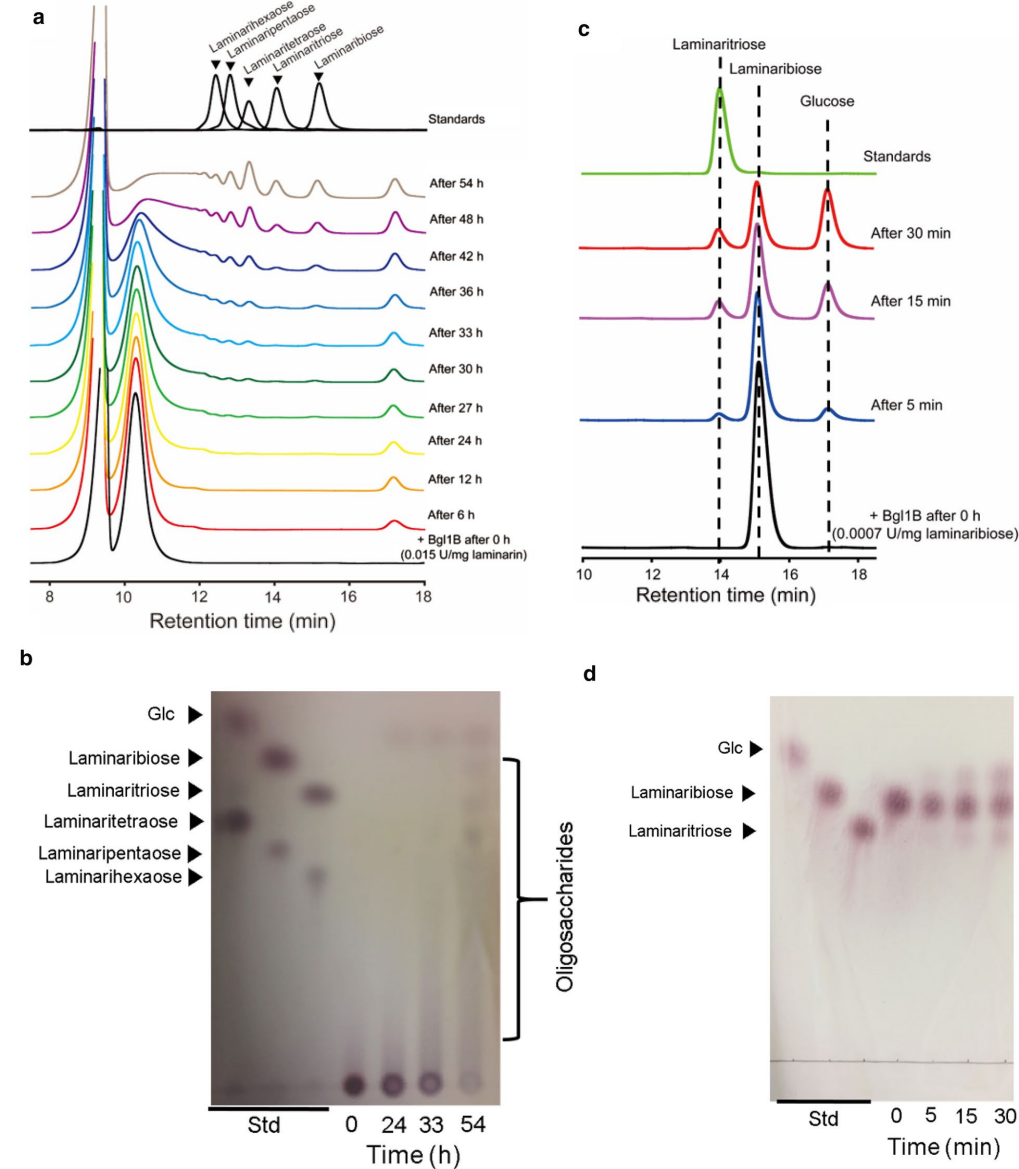
Transglycosylation activity of Bgl1B toward laminarin and laminaribiose as substrates. **a**, **c** HPLC and **b**, **d** TLC analyses of enzymatic reaction products toward laminarin and laminaribiose, respectively. The transglycosylation reactions were performed with 0.015 U mg^−1^ laminarin and 0.0007 U mg^−1^ laminaribiose, respectively, at 40 °C in 20 mM sodium phosphate (pH 6.0). Std, standard; Glc, glucose

When laminarin and laminaribiose were subjected to low enzyme concentrations of Bgl1B, the transglycosylation activity of Bgl1B was predominantly exhibited by producing various laminarioligosaccharides from laminarin and producing laminaritriose from laminaribiose (Fig. 6). After 0.2% (w/v) of laminarin was incubated with 0.03 U of Bgl1B, the reaction products obtained over a period of 54 h were subjected to HPLC (Fig. 6a) and TLC analyses (Fig. 6b). As the reaction progressed for 24 h, only glucose was increasingly produced by Bgl1B (Fig. 6a, b). These results were similar to those of laminarin hydrolysis with 1.5 U mg^−1^ laminarin (Fig. 5b). However, as the reaction further progressed from 27 to 33 h, glucose did not significantly increase, which is different from the results of the hydrolysis of laminarin (Fig. 5b). Instead, low-degree of polymerization (DP) oligosaccharides such as DP2, DP3, and DP4 began to appear. In addition, when the reaction was further prolonged over 33 h, oligosaccharides were produced as the main products by possibly a transglycosylation reaction (Fig. 6a, b). To further examine the transglycosylation activity of Bgl1B, 0.3% (w/v) of laminaribiose was used as a substrate for 0.002 U of Bgl1B. From laminaribiose, Bgl1B produced DP3 and glucose (Fig. 6c, d). These results suggested that Bgl1B uses laminaribiose as a building block for its transglycosylation reaction to produce laminaritriose. Therefore, in the transglycosylation reaction by Bgl1B, laminaribiose and probably laminarioligosaccharides can serve as both a glucose donor and acceptor, leading to synthesis of oligosaccharides with various DPs.

## Discussion

In this study, we have found and characterized the first and unique β-glucosidase, Bgl1B, from *S. degradans* 2-40^T^, a marine bacterium. Bgl1B was found to be a β-glucosidase belonging to GH1 (Additional file 2: Figure S1) and also to possess strong hydrolyzing activity toward laminaribiose having a β-1,3-glycosidic linkage (Table 2 and Fig. 4b). Currently, the CAZy database contains a total of 145 GH families, and of them, β-glucosidases can be found in GH1, GH3, GH5, GH9, GH30, and GH116. Although there are several studies on β-glucosidases from GH1, originating from various microorganisms [12], a β-glucosidase from *S. degradans* 2-40^T^ has not been reported yet.

In this study, although Bgl1B showed broad specificity toward various disaccharides having β-1,4- β-1,6-, or β-1,3-glycosidic linkage, such as cellobiose, lactose, agarobiose, gentiobiose, and laminaribiose (Fig. 4a, b), a significantly higher specific activity was shown toward laminaribiose, which possesses a β-1,3-glycosidic linkage (Table 2). Bgl1B showed higher specific activity for laminaribiose than that for other substrates tested in this study. However, most of other reported β-glucosidases belonging to GH1 showed higher or similar substrate specificity for cellobiose than that for laminaribiose (Additional file 4: Table S2). Taken together, to our knowledge, Bgl1B from *S. degradans* 2-40^T^ is the unique enzyme having significantly high laminaribiose-hydrolyzing activity unlike other several bacterial β-glucosidases belonging GH1.

Laminarin, the polysaccharide of laminaribiose, is one of the major storage carbohydrates in brown macroalgae. Brown macroalgae are considered a potential bioresource for the production of biofuels and bio-based chemicals [6]. Laminarin is composed of β-1,3-glucans in the backbone and β-1,6-glucans in the branches [3]. To date, similar to cellulose depolymerization, laminarin is degraded to oligosaccharides with low DPs or laminaribiose by β-1,3- and β-1,6-glucanases, and laminaribiose is then hydrolyzed into glucose by β-glucosidases. There have been many studies on laminarinases (i.e., β-1,3-glucanases) from a variety of marine organisms [13–15]. For example, Gly5M, which was reported as an endo-type β-glucanase from *S. degradans* 2-40^T^, can efficiently hydrolyze laminarin into various oligosaccharides with a small amount of glucose [8]. However, these reported laminarinases including Gly5M did not show laminaribiase activity. Until now, laminaribiases have been only reported from *Aplysia kurodai* [16] and *Thermotoga neapolitana* [17]. Compared to GH3 β-glucosidase from *T. neapolitana*, the *K*_m_ value of Bgl1B studied here was approximately 10 times lower, which indicates strong affinity of Bgl1B toward laminaribiose.

In this study, Bgl1B was found to also hydrolyze laminarin into glucose with a high conversion yield (e.g., 75%) as an exo-type β-1,3-glucanase (Fig. 5a). This high exo-type β-1,3-glucanase activity of Bgl1B toward laminarin is important for the saccharification of brown macroalgal biomass because the enzyme cost for saccharifying biomass accounts for a large part in producing biofuels and bio-based products [18]. Regarding the hydrolysis of laminarin, most studies have focused on the hydrolysis of laminarin into oligosaccharides by endo- and/or exo-β-glucanases (i.e., laminarinases) [8–10], but there has been no report on laminarin depolymerization using β-glucosidases (i.e., laminaribiases). Therefore, Bgl1B has great potential in industrial production of biofuels and bio-based products from brown macroalgal biomass.

In addition to the high specific activity and broad substrate specificity of Bgl1B, it was also found to be psychrophilic. Bgl1B showed high relative activity in a broad range of temperature such as 2–20 °C (Fig. 3b). Among characterized β-glucosidases in GH1 (Additional file 4: Table S2), only two glucosidases showed psychrophilicity. The β-glucosidase from *Micrococcus antarcticus*, a psychrophilic microorganism, has the optimal temperature at 25 °C, and showed a relative activity of 57% even at 10 °C [19]. Also, a β-glucosidase from *Bifidobacterium breve* showed a relative activity of 95% at 20 °C [20]. Except these two enzymes, most of characterized β-glucosidases in GH1 showed a relative activity of less than 50% even at 30 °C (Additional file 4: Table S2). Psychrophilic enzymes can have industrial potential since they can be used in energy-efficient processes at low temperature [21].

In addition to the β-glucosidase and exo-type β-1,3-glucanase activities of Bgl1B, it also exhibited transglycosylation activity. A few other β-glucosidases in GH1 are also known to have transglycosylation activity [22]. In this study, Bgl1B produced laminarioligosaccharides with various DPs from laminarin, and laminaritriose from laminaribiose through its transglycosylation reaction (Fig. 6). Transglycosylation activity is known to increase at high ratios of substrate to enzyme concentration [23, 24]. In this study, to produce oligosaccharides from laminarin and laminaribiose through the transglycosylation activity of Bgl1B, we performed enzymatic reactions under lower concentrations of Bgl1B (i.e., 0.015 U mg^−1^ laminarin and 0.0007 U mg^−1^ laminaribiose) than what was used for the hydrolysis reactions. These reactions yielded laminarioligosaccharides with various DPs and laminaritriose from laminarin (Fig. 6a, b) and laminaribiose (Fig. 6c, d), respectively. In this transglycosylation reaction using Bgl1B with laminarin, laminarioligosaccharides and glucose released from the hydrolysis of laminarin may have served as the substrates for transglycosylation, leading to the production of oligosaccharides having DPs higher than that of their substrates. It is well known that laminarioligosaccharides have various physiological functions [25].

## Conclusions

Laminarin, a polysaccharide composed of glucose, can be an important resource for glucose which can be easily converted to biofuels and bio-based chemicals by microorganisms. In effective use of laminarin, Bgl1B from *S. degradans* 2-40^T^ is a unique β-glucosidase in GH1. First, Bgl1B has high substrate specificity toward laminaribiose. Second, although Bgl1B is an intracellular β-glucosidase of *S. degradans* 2-40^T^, Bgl1B can hydrolyze laminarin into glucose with a high conversion yield. Third, Bgl1B has high enzymatic activity at low temperatures, such as below 20 °C. To our knowledge, this is the first report of a β-glucosidase in GH1, which can efficiently hydrolyze laminarin into glucose as a sole product. In addition, Bgl1B can produce laminarioligosaccharides with various DPs by transglycosylation, and these oligosaccharides are known to possess various biological activities. Taken together, Bgl1B can be used for producing not only biofuels and bio-based products but also high-value oligosaccharides from laminarin.

## Methods

### Cloning and expression of *bgl1B* gene

*S. degradans* 2-40^T^ (ATCC 43961) was grown in a sea salt minimal medium [2.3 g synthetic sea salt (Aquarium Systems, Mentor, OH), 20 mmol Tris–HCl, 2 g glucose, 1 g yeast extract, and 0.5 g ammonium chloride per liter] at 30 °C for 12 h. Genomic DNA of *S. degradans* was extracted using a commercial DNA isolation kit (Bioneer, Daejeon, Korea). The *bgl1B* gene was amplified from the genomic DNA by a polymerase chain reaction (PCR) using the following primers: forward primer, 5′-ATACATATGAATAGACTTACACTACCGCCTTCTTCTCGT-3′ and reverse primer, 5′-ATAGCGGCCGCGCTCCTACTCGAGACAAACTCAGCAAATGC-3′. A poly-6-histidine residue was added to the C terminus of the protein to purify the protein by affinity chromatography (GE Healthcare, Piscataway, NJ). PCR product and pET21a (Novagen, Darmstadt, Germany) were digested using NdeI and NotI, and were ligated to construct pET21a–Bgl1B plasmid. Recombinant *E. coli* BL21(DE3) harboring the pET21a–Bgl1B plasmid was grown in Luria–Bertani (LB) broth containing 50 mg L^−1^ of ampicillin at 37 °C and 200 rpm until the optical density of culture broth measured at 600 nm reached 0.5. Overexpression of the enzyme was induced by 0.5 mM isopropyl-β-d-thiogalactopyranoside (IPTG) (Sigma, St. Louis, MO) at 16 °C for 16 h.

### Purification of the recombinant Bgl1B

To obtain the recombinant Bgl1B, recombinant cells were harvested by centrifugation at 4000×*g* for 30 min at 4 °C. The cell pellet was resuspended into a lysis buffer (20 mM Tris–HCl, pH 7.4), disrupted by ultrasonication, and centrifuged at 8000×*g* for 30 min at 4 °C. The supernatant was loaded onto a HisTrap column (GE Healthcare, Piscataway, NJ), which was already equilibrated with a binding buffer (20 mM Tris–HCl, 0.5 M NaCl, 20 mM imidazole, pH 7.4) for 15 min at 1 mL min^−1^. To release the loaded protein from the HisTrap column, 15 mL of elution buffers with different imidazole concentrations ranging from 20 to 500 mM was eluted. The eluted recombinant Bgl1B was desalted and concentrated using an Amicon ultracentrifugal filter unit (MW cutoff value of 30,000; UFC903024, Millipore, Billerica, MA). The purified recombinant Bgl1B was analyzed by 8% (w/v) sodium dodecyl sulfate polyacrylamide gel electrophoresis (SDS-PAGE). The protein concentration was quantified using bicinchoninic acid protein assay kit (Thermo Fisher Scientific, Waltham, MA) and bovine serum albumin (Sigma) was used as a protein standard.

### Enzyme assay of Bgl1B

To examine the enzymatic activity of recombinant Bgl1B, 2.0 nmol protein was incubated in 1 mL of 20 mM sodium phosphate buffer (pH 6.0) containing 0.1% (w/v) cellobiose (Sigma) at 40 °C for 15 min for the enzymatic hydrolysis of cellobiose by Bgl1B. The enzymatic reaction was stopped by incubating the reaction mixture in boiling water for 1 min. The enzymatic activity was measured by quantifying the amount of glucose produced from the hydrolysis of cellobiose using high-performance liquid chromatography (HPLC). One unit (U) of Bgl1B was defined as the amount of enzyme required to produce 1 μmol of glucose per min under the enzymatic reaction conditions described above.

### Analysis of enzymatic reaction products of Bgl1B

The enzymatic reaction products of Bgl1B were analyzed by thin-layer chromatography (TLC). One microliter aliquot from each reaction mixture was loaded onto silica gel 60 TLC plates (Merck, Darmstadt, Germany), and developed using *n*-butanol–acetic acid–water (3:2:2, v/v/v). The plate loaded with the sample was visualized using 10% (v/v) H_2_SO_4_ and 0.2% (w/v) naphthoresorcinol in ethanol. The reaction products of Bgl1B were also analyzed by an Agilent 1100 HPLC system (Agilent Technologies, Santa Clara, CA) equipped with a gel permeation column (KS-802; Shodex, Tokyo, Japan) and a refractive index detector (Agilent) using distilled water as the mobile phase with a flow rate of 0.5 mL min^−1^ at 80 °C.

### Screening for substrate specificity of Bgl1B

To examine the substrate specificity of Bgl1B, 9 different disaccharides and agarotriose, which possess various types of glycosidic bonds, were tested. The first substrate group consisted of terrestrial biomass-derived disaccharides, namely cellobiose (formed with two units of d-glucose with a β-1,4-glycosidic linkage), lactose (formed with d-galactose and d-glucose units with a β-1,4-glycosidic linkage), gentiobiose (formed with two units of d-glucose with a β-1,6-glycosidic linkage), sucrose (formed with d-glucose and d-fructose units with an α-1,2-glycosidic linkage), maltose (formed with two d-glucose units with an α-1,4-glycosidic linkage), and melibiose (formed with d-galactose and d-glucose units with an α-1,6-glycosidic linkage) (all purchased from Sigma). The second substrate group consisted of marine biomass-derived disaccharides, namely laminaribiose (formed with two units of d-glucose with a β-1,3-glycosidic linkage) (Megazyme, Wicklow, Ireland), agarobiose (formed with d-galactose and 3,6-anhydro-l-galactose (AHG) units with a β-1,4-glycosidic linkage), neoagarobiose (formed with AHG and d-galactose units with an α-1,3-glycosidic linkage), and agarotriose (formed with d-galactose, AHG, and d-galactose with β-1,4- and α-1,3-glycosidic linkages alternately) (Beijing Ad Hoc International Technologies, Beijing, China). Agarobiose and neoagarobiose were produced in-house as described previously with slight modifications [26]. To screen the substrate specificity of Bgl1B, 0.6 U of enzyme was incubated at 40 °C for 1 h in 20 mM sodium phosphate buffer (pH 6.0) containing 0.2% (w/v) of each substrate.

### Enzyme characterization of Bgl1B

To determine the optimum pH for Bgl1B activity, 0.06 U of the enzyme was incubated at 40 °C for 15 min with 0.1% (w/v) cellobiose in various buffers with different pH values, such as 20 mM sodium acetate (pH 4.0–6.0), 20 mM sodium phosphate (pH 6.0–7.0), 20 mM Tris–HCl–NaCl (pH 7.0–9.0). To determine the optimal temperature of Bgl1B, 0.06 U of Bgl1B was incubated with 0.1% (w/v) cellobiose in 20 mM sodium phosphate buffer (pH 6.0) for 15 min at various temperatures ranging from 2 to 60 °C. To determine the thermostability of Bgl1B, 0.06 U of Bgl1B was pre-incubated at various temperatures ranging from 2 to 60 °C for 1 h prior to the enzymatic reaction with 0.1% (w/v) cellobiose in the same buffer.

To examine the effect of metal ions, namely Mg^2+^, Ca^2+^, Mn^2+^, Ni^2+^, Cu^2+^, Fe^2+^, and Co^2+^, on the enzymatic activity of Bgl1B, the reaction was performed in the presence of 10 mM of each metal ion with 0.1% (w/v) cellobiose in 20 mM sodium phosphate buffer (pH 6.0) at 40 °C for 15 min.

To determine the kinetic parameters of Bgl1B toward various substrates, namely laminaribiose, cellobiose, gentiobiose, lactose, and agarobiose, the enzymatic reactions were performed with different concentrations of each substrate ranging from 0.1 to 0.4% (w/v) under optimal pH and temperature determined as described earlier. The enzyme kinetic parameters of Bgl1B, *V*_max_, *K*_m_, and *k*_cat_ were determined from the Lineweaver–Burk plot.

To examine the mode of action of Bgl1B, 3 U of Bgl1B was incubated with 0.2% (w/v) laminarin (Tokyo Chemical Industry, Tokyo, Japan), which consists of β-1,3-glucan as the main backbone and β-1,6-glucan as the branches, and 0.2% (w/v) pustulan (InvivoGen, San Diego, CA), which consists of β-1,6-glucan, at 40 °C in 20 mM sodium phosphate (pH 6.0) for 24 and 12 h, respectively. To investigate the possible transglycosylation activity of Bgl1B, 0.03 and 0.002 U of Bgl1B was incubated with 0.2% (w/v) laminarin and 0.3% (w/v) laminaribiose at 40 °C in 20 mM sodium phosphate for 54 h and 30 min, respectively.

## Additional files

**Additional file 1: Table S1.** Kinetic parameters^a^ of Bgl1B in the hydrolysis of different types of substrates.

**Additional file 2: Figure S1.** The phylogenetic tree of Bgl1B and other characterized bacterial β-glucosidases in glycoside hydrolase family 1 (GH1). *Thermotoga neapolitana* DSM 4359 in GH3 was used to compare between β-glucosidases from GH1 and GH3. The nucleotide sequences of the enzymes were obtained from UniProt and the CAZy database, and aligned using RDP release 11 tool. The phylogenetic tree was drawn using the MEGA 7 program. The accession numbers are indicated after the microorganism names.

**Additional file 3: Figure S2.** TLC analysis for the hydrolysis of pustulan substrate using Bgl1B. The reaction was performed at 40 °C in 20 mM sodium phosphate buffer (pH 6.0) with 0.2% (w/v) pustulan. Abbreviations: Std, standard; Glc, glucose; −, substrate only; +, substrate incubated with 0.2 U of Bgl1B/mg of pustulan for 12 h.

**Additional file 4: Table S2.** Comparison of optimum conditions and substrate specificities of various characterized β-glucosidases (EC 3.2.1.21) in GH1.

## Abbreviations

GH: glycoside hydrolase family
DP: degree of polymerization
LB: Luria–Bertani
U: unit
IPTG: isopropyl-β-d-thiogalactopyranoside
SDS-PAGE: sodium dodecyl sulfate polyacrylamide gel electrophoresis
HPLC: high-performance liquid chromatography
TLC: thin-layer chromatography
AHG: 3,6-anhydro-l-galactose
Glc: glucose
Gal: galactose
Std: standard
