# Supplementary material for: A novel β-glucosidase from Saccharophagus degradans 2-40T for the efficient hydrolysis of laminarin from brown macroalgae
Source: Biotechnol Biofuels. 2018 Mar 14;11:64. doi: 10.1186/s13068-018-1059-2 (PMC5851131; doi:10.1186/s13068-018-1059-2)
Supplement: Supplementary file 1 — Additional file 1: Table S1. Kinetic parametersa of Bgl1B in the hydrolysis of different types of substrates. [file 13068_2018_1059_MOESM1_ESM.doc]

**Additional file 1**

**Table S1** Kinetic parameters^a^ of Bgl1B in the hydrolysis of different types of substrates

|  | Substrate | Glycosidic linkage | *V*_max_  (U^b^/mg protein) | *K_m_*  (mM) | *k_cat_*  (s^-1^) | *k_cat_*/*K_m_*  (mM^-1^ s^-1^) |
| --- | --- | --- | --- | --- | --- | --- |
| β-Glucosidase | Gentiobiose | β-1,6 | 1.1 x 10^0^ | 1.3 x 10^1^ | 8.8 x 10^-1^ | 6.6 x 10^-2^ |
| β-Galactosidase | Lactose | β-1,4 | 1.0 x 10^0^ | 2.0 x 10^1^ | 8.3 x 10^-1^ | 4.1 x 10^-2^ |
| β-Galactosidase | Agarobiose | β-1,4 | 3.1 x 10^-1^ | 6.9 x 10^1^ | 2.6 x 10^-1^ | 3.7 x 10^-3^ |

^a^Each value of parameter is the mean of experimental triplicates

^b^One unit (U) of Bgl1B was defined as the amount of enzyme required to produce 1 μmol of glucose per min from 0.1% (w/v) cellobiose in 20 mM sodium phosphate buffer (pH 6.0) at 40°C
