# Supplementary material for: A novel β-glucosidase from Saccharophagus degradans 2-40T for the efficient hydrolysis of laminarin from brown macroalgae
Source: Biotechnol Biofuels. 2018 Mar 14;11:64. doi: 10.1186/s13068-018-1059-2 (PMC5851131; doi:10.1186/s13068-018-1059-2)
Supplement: Supplementary file 2 — Additional file 2: Figure S1. The phylogenetic tree of Bgl1B and other characterized bacterial β-glucosidases in glycoside hydrolase family 1 (GH1). Thermotoga neapolitana DSM 4359 in GH3 was used to compare between β-glucosidases from GH1 and GH3. The nucleotide sequences of the enzymes were obtained from UniProt and the CAZy database, and aligned using RDP release 11 tool. The phylogenetic tree was drawn using the MEGA 7 program. The accession numbers are indicated after the microorganism names. [file 13068_2018_1059_MOESM2_ESM.doc]

**Additional file 2**


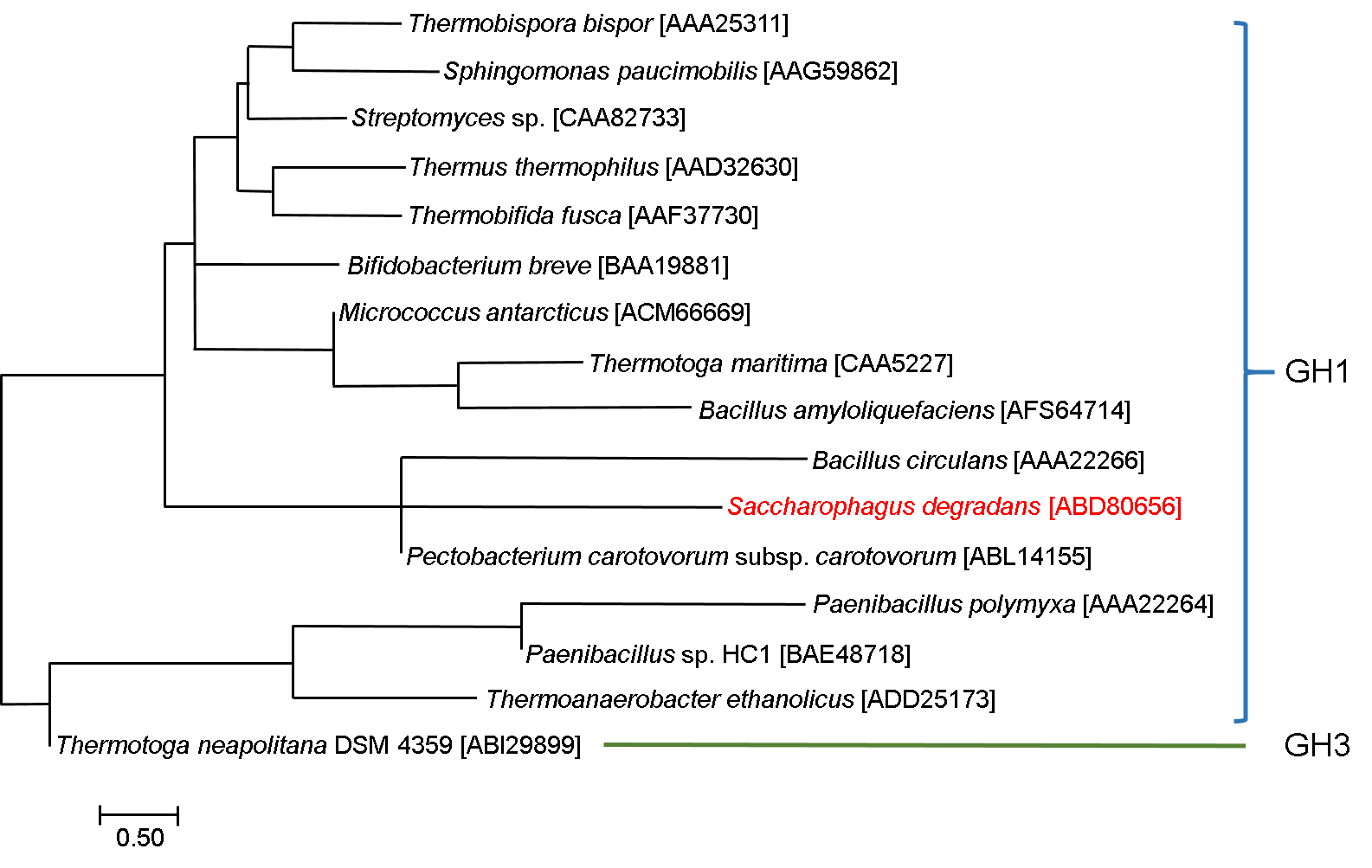


**Fig. S1** The phylogenetic tree of Bgl1B (indicated in red) and other characterized bacterial β-glucosidases in glycoside hydrolase family 1 (GH1). *Thermotoga neapolitana* DSM 4359 in GH3 was used to compare between β-glucosidases from GH1 and GH3. The nucleotide sequences of the enzymes were obtained from UniProt and the CAZy database, and aligned using RDP release 11 tool. The phylogenetic tree was drawn using the MEGA 7 program. The accession numbers are indicated after the microorganism names
