# Supplementary material for: A novel β-glucosidase from Saccharophagus degradans 2-40T for the efficient hydrolysis of laminarin from brown macroalgae
Source: Biotechnol Biofuels. 2018 Mar 14;11:64. doi: 10.1186/s13068-018-1059-2 (PMC5851131; doi:10.1186/s13068-018-1059-2)
Supplement: Supplementary file 3 — Additional file 3: Figure S2. TLC analysis for the hydrolysis of pustulan substrate using Bgl1B. The reaction was performed at 40 °C in 20 mM sodium phosphate buffer (pH 6.0) with 0.2% (w/v) pustulan. Abbreviations: Std, standard; Glc, glucose; −, substrate only; +, substrate incubated with 0.2 U of Bgl1B/mg of pustulan for 12 h. [file 13068_2018_1059_MOESM3_ESM.docx]

**Additional file 3**


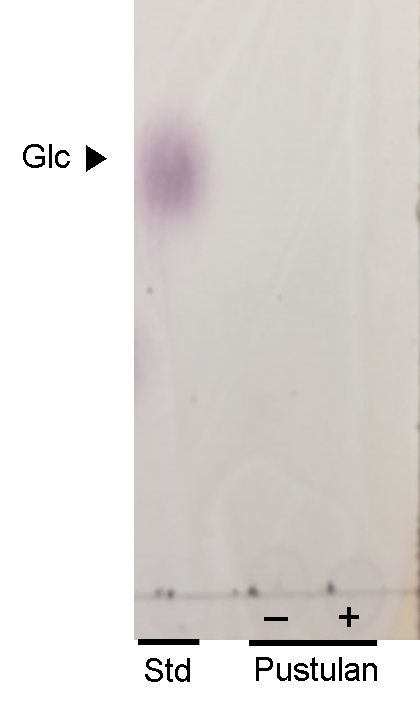


**Fig. S2** TLC analysis for the hydrolysis of pustulan substrate using Bgl1B. The reaction was performed at 40^°^C in 20 mM sodium phosphate buffer (pH 6.0) with 0.2% (w/v) pustulan. Abbreviations: Std, standard; Glc, glucose; −, substrate only; +, substrate incubated with 0.2 U of Bgl1B/mg of pustulan for 12 h
