# Supplementary material for: A novel β-glucosidase from Saccharophagus degradans 2-40T for the efficient hydrolysis of laminarin from brown macroalgae
Source: Biotechnol Biofuels. 2018 Mar 14;11:64. doi: 10.1186/s13068-018-1059-2 (PMC5851131; doi:10.1186/s13068-018-1059-2)
Supplement: Supplementary file 4 — Additional file 4: Table S2. Comparison of optimum conditions and substrate specificities of various characterized β-glucosidases (EC 3.2.1.21) in GH1. [file 13068_2018_1059_MOESM4_ESM.doc]

**Additional file 4**

**Table S2** Comparison of optimum conditions and substrate specificities of various characterized β-glucosidases (EC 3.2.1.21) in GH1

| Domain | Microorganism | Gene name | GenBank number | Optimal pH | Optimal temp. | Substrate specificity*^a^* | Reference |
| --- | --- | --- | --- | --- | --- | --- | --- |
| Archaea | *Pyrococcus horikoshii* | *PH0366* | BAA29440.1 | 6.0 | 100 | *p*-NPGlc, *p*-NPGal, salicin, cellobiose, laminaribiose | [1] |
| Eukaryota | *Humicola grisea* var. *thermoidea* | *bgl4* | BAA74958.1 | 6.0 | 55 | *p*-NPGlc, *p*-NPGal | [2] |
|  | *Phanerochaete chrysosporium* | *bgl1B* | BAE87009.1 | 6.5 | 30 | Cellobiose, *p*-NPGlc, *p*-NPGal | [3] |
|  | *Trichoderma reesei* | *bgl2* | BAA74959.1 | 6.0 | 40 | *p*-NPGlc, *p*-NPGal | [2] |
| Bacteria | *Bacillus amyloliquefaciens* ABBD | *bglZ* | JX422023.1 | NA*^b^* | NA | *p*-NPGlc | [4] |
|  | *Bacillus circulans* | *bglA* | AAA22266.1 | 7.0-8.0 | 37 | Cellobiose, *p*-NPGlc, *o*-NPGlc, lactose, salicin | [5] |
|  | *Bifidobacterium breve* | NA | BAA19881.1 | 5.5 | 45 | *p*-NPGlc, laminaribiose, sophorose, *p*-NPFuc, cellobiose, salicin, *p*-NPGal | [6] |
|  | *Halothermothrix orenii* | *bglA* | ACL70277.1 | 6.0-7.0 | 65-70 | Cellobiose, lactose | [7] |
|  | *Micrococcus antarcticus* | *bglU* | ACM66669.1 | 6.5 | 25 | Cellobiose, *p*-NPGlc, laminaribiose, sophorose, *p*-NPGal, gentiobiose | [8] |
|  | *Paenibacillus polymyxa* | *bglB* | AAA22264.1 | 7.0 | 37 | Cellobiose, cellodextrins | [9] |
|  | *Paenibacillus* sp. HC1 | *bglA* | BAE48718.1 | 7.0 | 37 | *p*-NPGlc, *p*-NPFuc, *p*-NPGal | [10] |
|  | *Pectobacterium carotovorum* subsp. *Carotovorum* | *celG* | ABL14155.1 | 5.0 | 40 | *p*-NPGlc, cellobiose, salicin, arbutin | [11] |
|  | *Saccharophagus degradans* | *bgl1B* | ABD80656.1 | 6.0 | 40 | Laminaribiose, cellobiose, gentiobiose, lactose, agarobiose | This study |
|  | *Sphingomonas paucimobilis* | *bgl1* | AAG59862.1 | 6.2 | 50 | *p*-NPGlc, *p*-NPGal, *p*-NPXyl, *o*-NPGlc, cellobiose (X), lactose (X) | [12] |
|  | *Streptomyces* sp. QM-B814 | *bgl3* | CAA82733.1 | 6.5 | 50 | Cellotetraose, cellobiose, cellotriose, sophorose, lactose, laminaribiose, *p*-NPGlc, salicin | [13] |
|  | *Thermoanaerobacter ethanolicus* | *bglA* | ADD25173.1 | 7.0 | 80 | *p*-NPGlc, daidzin, genistin | [14] |
|  | *Thermobifida fusca* | *bglC* | AAF37730.1 | 7.0 | 50 | Cellobiose, cellotetraose, cellotriose, sophorose, *p*-NPGlc, *p*-NPGal | [15] |
|  | *Thermobispora bispora* | *bglB* | AAA25311.1 | 6.2 | 60 | Cellobiose, esculin, *p*-NPGlc | [16] |
|  | *Thermotoga maritima* | *bglA* | CAA52276.1 | 6.2 | 70 | *p*-NPGlc, *p*-NPFuc, *p*-NPGal, cellobiose, lactose, salicin, arbutin | [17] |
|  | *Thermus thermophilus* | NA | AAD32630.2 | 5.4 | 90 | *p*-NPFuc, *o*-NPGal, *p*-NPGlc, *p*-*p*-NPGal | [18] |

The above information was gathered from the CAZy database (http://www.cazy.org/) and the Uniprot (http://www.uniprot.org/)

*^a^*Substrates are listed in order of high substrate specificity for each enzyme

*^b^*NA, not applicable

**Additional References**

1. Matsui I, Sakai Y, Matsui E, Kikuchi H, Kawarabayasi Y, Honda K. Novel substrate specificity of a membrane‐bound β‐glycosidase from the hyperthermophilic archaeon *Pyrococcus horikoshii*. FEBS lett. 2000;467:195-200.

2. Takashima S, Nakamura A, Hidaka M, Masaki H, Uozumi T. Molecular cloning and expression of the novel fungal β-glucosidase genes from *Humicola grisea* and *Trichoderma reesei*. J Biochem. 1999;125:728-736.

3. Tsukada T, Igarashi K, Yoshida M, Samejima M. Molecular cloning and characterization of two intracellular β-glucosidases belonging to glycoside hydrolase family 1 from the basidiomycete *Phanerochaete chrysosporium*. Appl Microbiol Biotechnol. 2006;73:807-814.

4. Kurniasih SD, Alfi A, Natalia D, Radjasa OK, Nurachman Z. Construction of individual, fused, and co-expressed proteins of endoglucanase and β-glucosidase for hydrolyzing sugarcane bagasse. Microbiol Res. 2014;169:725-732.

5. Paavilainen S, Hellman J, Korpela T. Purification, characterization, gene cloning, and sequencing of a new beta-glucosidase from *Bacillus circulans* subsp. *alkalophilus*. Appl Environ Microbiol. 1993;59:927-932.

6. Nunoura N, Ohdan K, Tanaka K, Tamaki H, Yano T, Inui M, Yukawa H, Yamamoto K, Kumagai H. Cloning and nucleotide sequence of the β-D-glucosidase gene from *Bifidobacterium breve* clb, and expression of β-D-glucosidase activity in *Escherichia coli*. Biosci Biotechnol Biochem. 1996;60:2011-2018.

7. Hassan N, Nguyen T-H, Intanon M, Kori LD, Patel BK, Haltrich D, Divne C, Tan TC. Biochemical and structural characterization of a thermostable β-glucosidase from *Halothermothrix orenii* for galacto-oligosaccharide synthesis. Appl Microbiol Biotechnol. 2015;99:1731-1744.

8. Fan H-X, Miao L-L, Liu Y, Liu H-C, Liu Z-P. Gene cloning and characterization of a cold-adapted β-glucosidase belonging to glycosyl hydrolase family 1 from a psychrotolerant bacterium *Micrococcus antarcticus*. Enzyme Microb Technol. 2011;49:94-99.

9. Isorna P, Polaina J, Latorre-García L, Cañada FJ, González B, Sanz-Aparicio J. Crystal structures of *Paenibacillus polymyxa* β-glucosidase B complexes reveal the molecular basis of substrate specificity and give new insights into the catalytic machinery of family I glycosidases. J Mol Biol. 2007;371:1204-1218.

10. Harada KM, Tanaka K, Fukuda Y, Hashimoto W, Murata K. Degradation of rice bran hemicellulose by *Paenibacillus* sp. strain HC1: gene cloning, characterization and function of β-D-glucosidase as an enzyme involved in degradation. Arch Microbiol. 2005;184:215-224.

11. Hong SY, Cho KM, Math RK, Kim YH, Hong SJ, Cho YU, Kim H, Yun HD. Characterization of the recombinant cellobiase from *celG* gene in the beta-glucoside utilization gene operon of *Pectobacterium carotovorum* subsp. *carotovorum* LY34. J Mol Catal B Enzym. 2007;47:91-98.

12. Marques AR, Coutinho PM, Videira P, Fialho AM, Isabel S-C. *Sphingomonas paucimobilis* beta-glucosidase Bgl1: a member of a new bacterial subfamily in glycoside hydrolase family 1. Biochem J. 2003;370:793-804.

13. Perez‐Pons JA, Cayetano A, Rebordosa X, Lloberas J, Guasch A, Querol E. A β‐glucosidase gene (*bgl3*) from *Streptomyces* sp. strain QM‐B814. FEBS J. 1994;223:557-565.

14. Song X, Xue Y, Wang Q, Wu X. Comparison of three thermostable β-glucosidases for application in the hydrolysis of soybean isoflavone glycosides. J Agric Food Chem. 2011;59:1954-1961.

15. Spiridonov NA, Wilson DB. Cloning and biochemical characterization of BglC, a β-glucosidase from the cellulolytic actinomycete *Thermobifida fusca*. Curr Microbiol. 2001;42:295-301.

16. Wright RM, Yablonsky MD, Shalita ZP, Goyal A, Eveleigh D. Cloning, characterization, and nucleotide sequence of a gene encoding *Microbispora bispora* BglB, a thermostable beta-glucosidase expressed in *Escherichia coli*. Appl Environ Microbiol. 1992;58:3455-3465.

17. Gabelsberger J, Liebl W, Schleifer K-H. Cloning and characterization of β-galactoside and β-glucoside hydrolysing enzymes of *Thermotoga maritima*. FEMS Microbiol Lett. 1993;109:131-137.

18. Nam E, Kim M, Lee H, Ahn J. β-Glycosidase of *Thermus thermophilus* KNOUC202: gene and biochemical properties of the enzyme expressed in *Escherichia coli*. Appl Biochem Microbiol. 2010;46:515-524.
